# The occurrence and ecology of microbial chain elongation of carboxylates in soils

**DOI:** 10.1038/s41396-021-00893-2

**Published:** 2021-02-08

**Authors:** Sayalee Joshi, Aide Robles, Samuel Aguiar, Anca G. Delgado

**Affiliations:** 1 School of Sustainable Engineering and the Built Environment, Arizona State University, Tempe, AZ USA; 2 Biodesign Swette Center for Environmental Biotechnology, Arizona State University, Tempe, AZ USA; 3 Engineering Research Center for Bio-mediated and Bio-inspired Geotechnics (CBBG), Arizona State University, Tempe, AZ USA; 4 Department of Civil and Environmental Engineering, University of Illinois at Urbana-Champaign, Urbana, IL USA

**Keywords:** Soil microbiology, Microbial ecology

## Abstract

Chain elongation is a growth-dependent anaerobic metabolism that combines acetate and ethanol into butyrate, hexanoate, and octanoate. While the model microorganism for chain elongation, *Clostridium kluyveri*, was isolated from a saturated soil sample in the 1940s, chain elongation has remained unexplored in soil environments. During soil fermentative events, simple carboxylates and alcohols can transiently accumulate up to low mM concentrations, suggesting in situ possibility of microbial chain elongation. Here, we examined the occurrence and microbial ecology of chain elongation in four soil types in microcosms and enrichments amended with chain elongation substrates. All soils showed evidence of chain elongation activity with several days of incubation at high (100 mM) and environmentally relevant (2.5 mM) concentrations of acetate and ethanol. Three soils showed substantial activity in soil microcosms with high substrate concentrations, converting 58% or more of the added carbon as acetate and ethanol to butyrate, butanol, and hexanoate. Semi-batch enrichment yielded hexanoate and octanoate as the most elongated products and microbial communities predominated by *C. kluyveri* and other *Firmicutes* genera not known to undergo chain elongation. Collectively, these results strongly suggest a niche for chain elongation in anaerobic soils that should not be overlooked in soil microbial ecology studies.

## Introduction

Microbial chain elongation is an anaerobic metabolism in which microorganisms grow and get energy by combining carboxylates, such as acetate (C2), with more reduced compounds, such as ethanol (C2), producing longer-chain carboxylates (typically C4–C8) [1, 2]. A connection between microbial chain elongation and soils was established more than 150 years ago. Béchamp [3] documented production of a high concentration of hexanoate (C6) in a bottle kept closed for months containing an aqueous ethanol solution with chalk (and soil microorganisms) collected from a dry riverbed. Work in the 1930s with freshwater and marine muds (including a canal mud from Delft, The Netherlands) later produced several isolates of a novel species, *Clostridium kluyveri* [1], the model microorganism for chain elongation. For *C. kluyveri*, microbial chain elongation of carboxylates is its only growth-dependent metabolism [1, 4, 5]. When fed with acetate and ethanol, *C. kluyveri* produces butyrate (C4), hexanoate (or caproate), and H_2_ as metabolites [6]; propionate (C3) and ethanol are converted to the odd-numbered carboxylates, valerate (C5), and heptanoate (C7) [7].

Carboxylate chain elongation by *C. kluyveri* is achieved via reverse β-oxidation [8], resulting in a two-C elongation per cycle (Eqs. 1–2). Running β-oxidation in the reserve direction is thermodynamically favorable [9] but requires certain environmental conditions, such as the presence of energy-rich, reduced compounds like ethanol, and a high enough H_2_ partial pressure (10^−1^ kPa) to prevent (forward) β-oxidation [10]. In chain elongation, the carboxylate is the electron acceptor and the more reduced compound (e.g., ethanol) is the electron donor [2]. As seen in Eqs. 1 and 2, the elongations reactions are thermodynamically favorable, yielding a similar Gibbs-free energy [11] per elongation step:

1 $${{{{{\mathrm{CH}}_{3}}{{\mathrm{COO}}^{-}}}}\atop{\mathrm{Acetate}}} + {{{\mathrm{CH}}_{3}{\mathrm{CH}}_{2}{\mathrm{OH}}}\atop{\mathrm{Ethanol}}} \to 	{{{\mathrm{CH}}_{3}{\mathrm{CH}}_{2}{\mathrm{CH}}_{2}{\mathrm{COO}}^ {-}}\atop{\mathrm{Butyrate}}} + {\mathrm{H}}_{2}{\mathrm{O}};\,{\Delta} {\mathrm{G}}^{\circ \prime} \\ 	= - 38.8\frac{{{\mathrm{kJ}}}}{{{\mathrm{reaction}}}}$$

2 $${{{\mathrm{CH}}_{3}{\mathrm{CH}}_{2}{\mathrm{CH}}_{2}{\mathrm{COO}}^ {-}}\atop{\mathrm{Butyrate}}} + {{{\mathrm{CH}}_{3}{\mathrm{CH}}_{2}{\mathrm{OH}}}\atop{\mathrm{Ethanol}}} \to {{{\mathrm{CH}}_{3}{\mathrm{CH}}_{2}{\mathrm{CH}}_{2}{\mathrm{CH}}_{2}{\mathrm{CH}}_{2}{\mathrm{COO}}^ {-}}\atop{\mathrm{Hexanoate}}} \\ + {\mathrm{H}}_{2}{\mathrm{O}};\,{\Delta} {\mathrm{G}}^{\circ \prime } = - 38.7\frac{{{\mathrm{kJ}}}}{{{\mathrm{reaction}}}}$$

H_2_ production, shown in Eq. 3, is a result of partial ethanol oxidation by ferredoxin-dependent hydrogenases and yields ATP and NADH for the microorganisms [8, 10]:

3 $${{{\mathrm{CH}}_{3}{\mathrm{CH}}_{2}{\mathrm{OH}}}\atop{\mathrm{Ethanol}}} +\, {\mathrm{H}}_{2}{\mathrm{O}} \to {{{\mathrm{CH}}_{3}{\mathrm{COO}}^ {-}}\atop{\mathrm{Acetate}}} +\, {\mathrm{H}}^ {+} + 2\,{\mathrm{H}}_2;\,{\Delta} {\mathrm{G}}^{\circ \prime } = + 9.7\frac{{{\mathrm{kJ}}}}{{{\mathrm{reaction}}}}$$

Given that Eq. 3 is endergonic, it must be coupled with the reactions shown in Eq. 1 and/or 2 for the net reaction to be thermodynamically favorable.

Since the isolation of *C. kluyveri*, work on microbial chain elongation has developed based on two trajectories. Early research focused on microbiological characterizations and resolving the biochemical pathways involved in producing and oxidizing fatty acids in *C. kluyveri* [7, 12–14]. A more recent focus exploits chain elongation by microbial communities containing *C. kluyveri* or other members of *Firmicutes* for production of biochemicals and biofuels [15, 16]. The latter has gained substantial momentum in engineering bio- [17, 18] and bioelectrochemical-reactors [19, 20] for conversion of various waste streams (i.e., food waste, municipal solid waste, agriculture waste, and syngas) into hexanoate and octanoate (C8), high-value medium-chain carboxylates. To that end, samples from natural and engineered environments have been used as bioreactor inocula and sources of chain-elongating microorganisms, including anaerobic digestor sludges, process wastewaters, rumen contents, and herbivore feces [18, 21].

Like municipal or agriculture waste streams, anaerobic soils and sediments often contain an abundance of biodegradable organic compounds. Under anaerobic conditions and with limited availability of electron acceptors, biodegradation of complex organic compounds is described by the anaerobic food web, a cascade of hydrolytic, fermentative, and methanogenic reactions involving a complex microbial community [22]. Transient fermentative accumulation of short-chain carboxylates and simple alcohols has been detected in anaerobic soils up to mM concentrations [23–25]. In top soils, short-chain carboxylates are also exudates from the roots of plants [26]. Given that microorganisms capable of chain elongation utilize carboxylates and simple alcohols as substrates, we hypothesized that microbial chain elongation could be a likely metabolic pathway in anaerobic soils. While sporadic accounts place *C. kluyveri* as part of the natural microbial communities of freshwater, brackish, or marine sediments [1, 5], the function and metabolism of *C. kluyveri* in its soil or sediment cradle remain unexplored. Furthermore, chain elongation is not considered in models for organic compounds biodegradation like the anaerobic food web [22].

In this study, we utilized four soil types collected from various depths with diverse biogeochemical characteristics in microcosms and enrichments in an effort to understand the occurrence of microbial chain elongation as a growth-supporting metabolism and its microbial ecology. We present evidence for microbial chain elongation as a substantial, but not previously considered, soil metabolism in microcosms and subsequent soil enrichments with implications for organic carbon dynamics and microbial ecology in anaerobic soil environments.

## Materials and methods

### Soil microcosms and conditions tested

Four soils were used in this study and are described in Table 1. The soils are referred to according to their city sampling location: Tempe, Bozeman, Lucas, and Goodyear. Tempe and Bozeman soils were collected using a hand trowel from a depth of 0.2–0.4 m below the ground surface. Lucas soil was excavated from a depth of 1.5 m. Goodyear soil was acquired through drill coring from a depth of 25–52 m below ground surface [27]. Equal parts of ~0.1 kg of soil cores from 25–52 m depths were homogenized to create composite Goodyear soil. All soils were thoroughly mixed in an anaerobic glove chamber before using them in microcosms.

**Table 1:** Description and characteristics of study soils.

Soil ID	Location	Description	Soil type	pH	Conductivity (µS cm^−1^)	Organic carbon (mg g^−1^ soil)
Tempe	Tempe, Arizona, USA	Top soil from an area covered with vegetation from 0.2–0.3 m below ground surface	Silty clay loam	7.8 ± 0.3	170 ± 30	37 ± 3
Bozeman	Bozeman, Montana, USA	Top soil from an area covered with vegetation from 0.2 to 0.4 m below ground surface	Silt loam	7.7 ± 0.2	180 ± 20	140 ± 7
Lucas	Lucas, Texas, USA	Deep soil from 1.5 m below ground surface	Clay	5.7 ± 0.5	80 ± 10	20 ± 3
Goodyear	Goodyear, Arizona, USA	Deep soil from 25 to 52 m below ground surface	Mix of loamy sand, sandy clay, and sand cores	7.6 ± 0.4	180 ± 10	5.7 ± 0.3

The data are averages with standard deviation of triplicate soil samples.

Duplicate soil microcosms were established in the anaerobic chamber in 250 mL glass serum bottles [28] according to Table 2. Microcosms consisted of 25 g of soil and 75 mL of reduced anaerobic mineral medium (1:3 soil to liquid ratio) as described previously [29]. The medium was buffered with 12.5 mM phosphate and had an initial pH of 7.5. 10 mM sodium 2-bromoethanesulfonate (BES), a methanogenesis inhibitor, was added at time zero in all microcosms. The substrate(s) concentrations and conditions tested are shown in Table 2. The initial concentration of H_2_ gas was 90 mM (nominal concentration). All microcosms were incubated in the dark at 30 °C and agitated at 125 rpm on a platform shaker.

**Table 2:** Experimental conditions tested in microcosms and enrichments with Tempe, Bozeman, Lucas, and Goodyear soils.

Microcosm condition	Soil used	Semi-batch cycle # incubation (days)				
		1	2	3	4	5
100 mM acetate + 100 mM ethanol^a,b^	All	14	8	6	9^c^	7^c^
100 mM ethanol^a^	All	14	6	43	7^c^	–
100 mM acetate + H_2_^a^	Lucas	14	8	6	16^c^	–
100 mM ethanol + H_2_^a^	Lucas	14	8	6	16^c^	–
140 mM acetate	All	14^c^	–	–	–	–
10 mM acetate + 10 mM ethanol	Tempe, Lucas	15	–	–	–	–
2.5 mM acetate + 2.5 mM ethanol	Tempe, Lucas	15	–	–	–	–
No substrates	All	14^c^	–	–	–	–

^a^Condition selected for enrichment in semi-batch cycles. A semi-batch cycle consisted of removing 25 mL microcosm slurry (one third of microcosm liquid) and replacing with 25 mL mineral medium containing acetate and/or ethanol at ~100 mM.

^b^Five cycles were completed for Tempe, Bozeman, and Lucas soils and four cycles were completed for Goodyear soil.

^c^Timepoint sampled for high-throughput DNA sequencing.

### Enrichments from microcosms in semi-batch cycles

The microcosms with 100 mM acetate + 100 mM ethanol, 100 mM ethanol, 100 mM acetate + H_2_, and 100 mM ethanol + H_2_ (Table 2) were subjected to an enrichment process in semi-batch cycles after the initial 14-day incubation (end of cycle 1). A semi-batch cycle consisted of removing 25 mL of microcosm slurry (one third of liquid) and replacing with 25 mL of fresh anaerobic medium with the corresponding substrate(s) at ~100 mM (Table 2). The concentration of H_2_ in the headspace was maintained at 90 mM (nominal concentration) by adding ultra-high purity H_2_ gas with a gastight syringe. BES was not added during the enrichment process. The enrichments were incubated in the dark at 30 °C according to the schedule from Table 2 and agitated at 125 rpm on a platform shaker.

### Chemical analytical methods

Initial soil pH and conductivity were measured in soil slurries (1:1 soil to DI water by weight) using an Oakton Multi-Parameter PCSTestr 35 probe (Vernon Hills, IL, USA). The probe was calibrated according to the manufacturer’s instructions using Oakton calibration standards. pH at time zero or end of a semi-batch cycle was measured directly on the microcosm slurry using a Sartorius pH bench top meter (Thermo Scientific, Waltham, MA, USA). The initial concentration of organic carbon in the soils was measured using a Shimadzu TOC-V SPH Total Carbon Analyzer (Columbia, MD, USA) with a solid state module (SSM-5000A) [30]. The calibration was performed using glucose (Acros Organics, NJ, USA) in a range of 0.5–25 mg C. The method minimum detection limit was 0.3 mg C kg^−1^ soil.

The concentrations of C2–C8 carboxylates, ethanol, butanol, and hexanol in microcosm liquid were analyzed using a high-performance liquid chromatograph (HPLC, Shimadzu LC-20AT) with a photodiode array detector at 210 nm and a refractive index detector [31–33]. The HPLC was equipped with an Aminex HPX-87H column (Bio-Rad, Hercules, CA, USA). The eluent was 2.5 mM H_2_SO_4_ at 0.6 mL min^−1^ for 30 min followed by 0.8 mL min^−1^ for another 90 min. The oven temperature was kept constant at 65 °C. The minimum detection limit for carboxylates was ≤0.04 mM and ≤0.1 mM for alcohols. The concentrations of substrates and metabolites in figures are shown in mM C to aid in following the carbon balance.

H_2_ and methane concentrations were analyzed from gas headspace samples using a gas chromatograph (GC, Shimadzu GC 2010) with a thermal conductivity detector (TCD) and a Carboxen 1010 PLOT column (Supelco, Bellefonte, PA, USA) [34]. The carrier gas was ultra-high purity argon with a constant pressure and flow rate of 42.3 kPa and 10 mL min^−1^, respectively. The temperature of the injector was 150 °C, the TCD temperature was 180 °C, and the current was set at 41 mA. The column temperature was initially held to 80 °C for 3 min, ramped to 160 °C at 50 °C min^−1^ and held for 1.5 min. The minimum detection limits for H_2_ and methane were 0.02 mM and 0.03 mM (gas concentrations), respectively. The total gas volume in the headspace of the microcosms was measured with a 50-mL frictionless syringe (Perfektum R matched numbered hypodermic syringes, Sigma Aldrich, St. Louis, MO, USA).

### DNA extraction and high-throughput DNA sequencing

Pellets for DNA extraction were obtained from 1.5 mL of microcosm soil slurry on day zero and at the end of incubation as noted in Table 2. Genomic DNA was extracted using a Power Soil DNA Isolation Kit (MO BIO Laboratories, Inc., Carlsbad, CA, USA). The DNA concentrations and purity were determined by measuring absorbance at wavelengths of 260 nm and 280 nm with a NanoDrop spectrophotometer (Rockland, DE, USA). Microbial community amplicon sequencing was performed using a MiSeq instrument (Illumina, Inc., San Diego, CA, USA) from the Center for Fundamental and Applied Microbiomics at the ASU KED Genomics Core Facility, Arizona State University, Tempe, AZ, USA. The primers were 515F (5′-GTGCCAGCMGCCGCGGTAA-3′) and 806R (5′-GGACTACHVGGGTWTCTAAT-3′) [35] for the V4 hyper-variable region of the 16S rRNA gene which captures Bacteria and Archaea [36]. Forward and reverse sequences (2 × 150 mode) were first paired (overlap ≥ 45 base-pairs) using PANDASeq [37]. The paired reads (average length 250 base pairs) were processed using the bioinformatics software Quantitative Insights into Microbial Ecology (QIIME 2, v. 2020.8) pipeline [38]. DADA2 was employed for detecting and correcting sequence data as a means for quality control and truncating each sequence at 151 base pairs to maintain a quality score of 25 or better. Taxonomy was assigned to amplicon sequence variants (ASVs) [39] using a pre-trained Naive Bayes classifier referencing SILVA database (v. 138) with a 99% identity threshold from 515F/806R region of sequences and the q2-feature-classifier plug in [40–42]. BLAST + consensus taxonomy classifier plug in was used to query the sequences against the National Center for Biotechnology (NCBI) database [43]. The raw sequence data were uploaded to NCBI and are available under project number PRJNA552606.

### Carbon and electron balances

Carbon and electron balances were performed for selected microcosm conditions to determine distribution of C and electrons from chain elongation substrates consumed to metabolites identified. The number of Cs per mol of compound are as follows: acetate, 2; ethanol, 2; butyrate, 4; butanol, 4; hexanoate, 6; hexanol, 6; and octanoate, 8. For electron balances, mmol of added substrates and metabolites were converted to millielectron equivalents (me^−^ equiv.). The number of me^−^ equiv. for each compound are as follows [11]: acetate, 8; ethanol, 12; butyrate, 20; butanol, 24; hexanoate, 32; hexanol, 36; and octanoate, 44. The C/electron balances were calculated by dividing mmol C or me^−^ equiv. of each metabolite at the end of a cycle by the total mmol C or me^−^ equiv. consumed from added substrate(s).

## Results and discussion

### Substantial microbial chain elongation activity in soils at high and environmentally relevant substrate concentrations

**Fig. 1:**
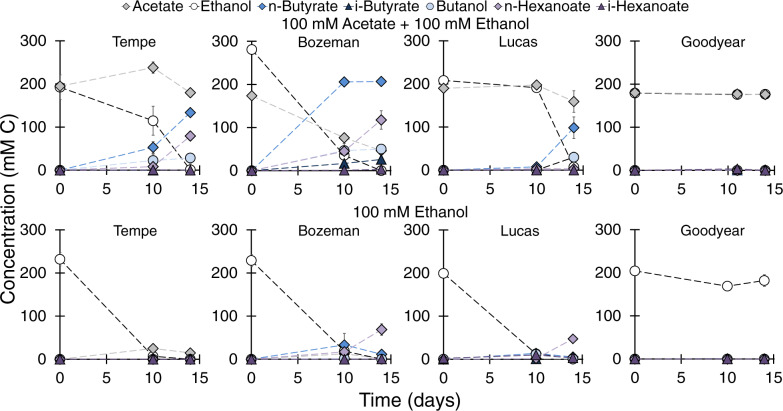
Microbial chain elongation activity in microcosms (25 g soil and 75 mL medium) with Tempe, Bozeman, Lucas, and Goodyear soils amended with 100 mM acetate and 100 mM ethanol or 100 mM ethanol. The data are averages with standard deviation of duplicate microcosms.

**Fig. 2:**
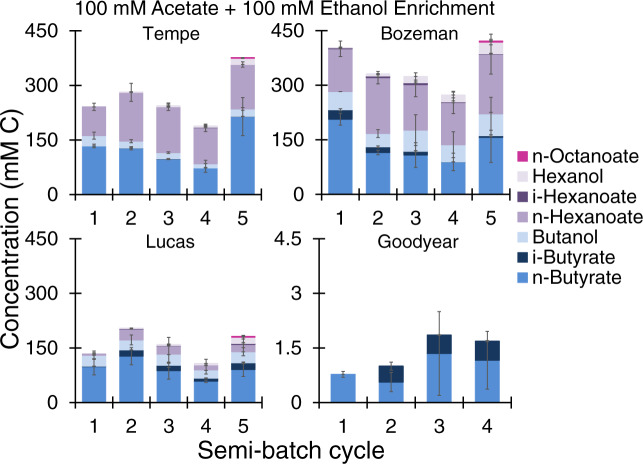
Metabolites during microbial chain elongation in soils initially fed with 100 mM acetate and 100 mM ethanol and subjected to semi-batch enrichment. A semi-batch cycle consisted of removing one third of microcosm liquid (25 mL) and replacing with 25 mL medium containing 100 mM acetate and 100 mM ethanol. The incubation time for each cycle (between 6–14 days) is shown in Table 2. The plotted carboxylates and alcohols are final measured metabolite concentrations at the end of each cycle before liquid was removed and medium with substrates was readded. The data are averages with standard deviation of duplicate microcosms.

**Fig. 3:**
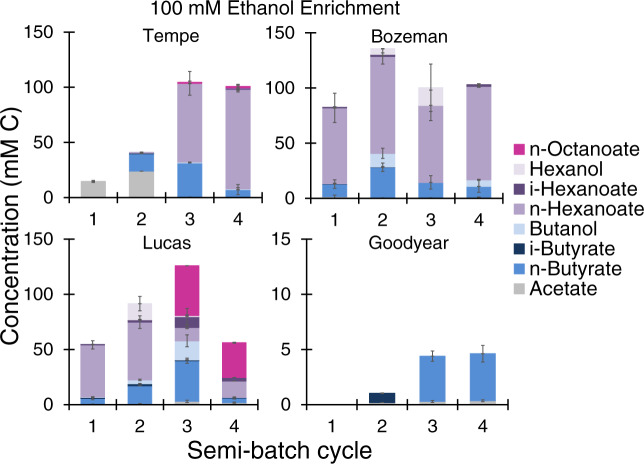
Metabolites during microbial chain elongation in soils initially fed with 100 mM ethanol and subjected to semi-batch enrichment. A semi-batch cycle consisted of removing one third of microcosm liquid (25 mL) and replacing with 25 mL medium containing 100 mM ethanol. The incubation time for each cycle (between 6–43 days) is shown in Table 2. The plotted carboxylates and alcohols are final measured metabolite concentrations at the end of each cycle before liquid was removed and medium with substrates was readded. The data are averages with standard deviation of duplicate microcosms.

The soils examined were chosen to capture different soil types (various proportions of silt, clay, and sand) with varying characteristics, including pH and endogenous organic carbon concentration (Table 1). Based on the sample collection depth, study soils were also expected to showcase varying levels of microbial activity (e.g., microbial activity typically decreases as a function of soil depth). All soils showed evidence of microbial chain elongation activity in at least one microcosm condition starting in cycle 1 (Figs. 1, 2–3, and S1 and S2, cycle 1). Microbial chain elongation of acetate and ethanol was the major metabolism occurring in Tempe, Bozeman, and Lucas soils, as evident by the high concentration of the metabolites. 58–117% of the consumed mmol C and 53–104% of the consumed me^−^ equiv. from substrates were recovered as C4–C6 carboxylates and alcohols by the end of cycle 1 (Table S1). We observed transient acetate accumulation as a metabolite in the ethanol microcosms (Fig. 1 and S2 (cycle 1)), possibly from ethanol oxidation (Eq. 2), which then initiated chain elongation to butyrate. Chain elongation activity was lowest but significant nonetheless in Goodyear soil (Table S1) where 0.78 ± 0.08 mM C *n*-butyrate was produced during cycle 1 (Fig. 2). Methane was absent in all conditions.

In control microcosms without added substrates, acetate was the only metabolite detected on day 14 of incubation (Fig. S3) at concentrations similar to those recorded in anaerobic incubations of forest [44, 45], bog [46], or paddy soils [47]. The absence of C4 or greater metabolites without added substrates and the C/electron balances (Table S1) corroborate the formation of butyrate, butanol, hexanoate, and hexanol from microbial chain elongation of added acetate and/or ethanol rather than processes involving the fermentation or oxidation of natural organic carbon of the soils. The soil microcosms with 140 mM acetate (no ethanol or H_2_) did not produce butyrate or hexanoate within 2 weeks of incubation (Fig. S3). These data substantiate that a more-reduced, organic C-containing compound must be present to serve as the electron donor for chain elongation activity to occur.

**Fig. 4:**
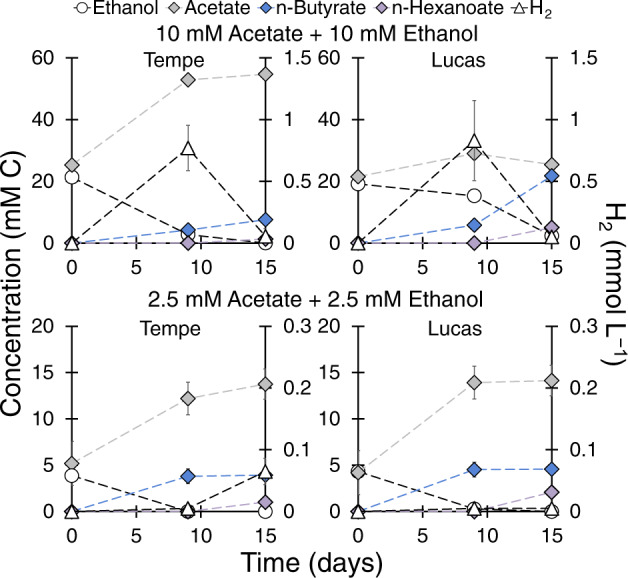
Microbial chain elongation of acetate and ethanol at lower substrate concentrations in Tempe and Lucas soil microcosms (25 g soil and 75 mL medium). The data are averages with standard deviation of duplicate microcosms.

We wondered if utilization of acetate and ethanol for microbial chain elongation is possible at lower, environmentally relevant concentrations observed during transient fermentative accumulation in soils. Figure 4 documents that the combination of acetate and ethanol at 10 mM each or 2.5 mM each triggers the use of acetate as a soil electron acceptor in chain elongation. Tempe and Lucas soils completely consumed the added ethanol, producing butyrate and ultimately hexanoate (Fig. 4). The microcosms in Fig. 4 showed a net accumulation of acetate higher than expected from ethanol oxidation (Eq. 3) or than observed in the control microcosms without added substrates (Fig. S3). A feasible pathway for acetate production in these microcosms is homoacetogenesis using HCO_3_^−^/CO_2_ as the electron acceptor [44, 48], which was conceivably activated by H_2_ released from chain elongation (Fig. 4).

### Subsequent enrichment augments microbial chain elongation to longer carboxylates

Microbial chain elongation activity continued in all soils in subsequent enrichment in semi-batch cycles (Figs. 2 and 3, and S1 and S2). In the acetate + ethanol soil enrichments, butyrate and hexanoate were the main metabolites produced, while *n*-octanoate (1.69 ± 0.01–2.94 ± 1.05 mM C) was also detected in Tempe and Bozeman soils during cycle 5 (Figs. 2 and S4). In Tempe, Bozeman, and Lucas soil enrichments, addition of ethanol (without acetate) augmented the production of more elongated carboxylates after two or three semi-batch cycles (Figs. 3 and S2). In Lucas soil, 5.66 ± 0.01 mM *n*-octanoate (45 mM C) was quantified by the end of cycle 3 when incubation was extended to 43 days (Fig. 3) and the pH was between 6.34 and 6.39 (Fig. S5). Accumulation of *n*-octanoate in Lucas soil enrichment during cycle 3 was possible as the undissociated *n*-octanoic acid concentration (0.18 mM) was three times less than the inhibitory concentration reported by Kucek et al. (0.63 mM undissociated *n*-octanoic acid) [49]. While hexanoate can be produced during acidogenic fermentation of complex biomass [15], octanoate is neither such metabolite nor a typical compound measured in soils. Production of octanoate is possible by diverting precursors of phospholipid biosynthesis but requires genetic engineering of microbial host strains [50]. Octanoate, however, is a metabolite of acetate and ethanol chain elongation. Its production was first documented in 2011 using a *C. kluyveri*-containing mixed culture enriched from a bioreactor treating brewery wastewater [15]. It has since been observed in other chain elongation works [49, 51–53]. Two factors contributed to conversion to longer metabolites in the ethanol soil enrichments (Fig. 3, Tempe, Bozeman, and Lucas soils). A first is the high concentration of the electron donor, ethanol, that facilitates from an energy stand point the use C2–C6 carboxylates as electron acceptors [15]. A second is the stoichiometric ratio of ethanol to acetate, which was higher in the microcosms with ethanol (Fig. 3, ≥9 mol ethanol: 1 mol acetate) compared to those in the microcosms with acetate and ethanol (Figs. 2, 1 mol ethanol: 1 mol acetate). To that extent, a number of studies showed that higher ethanol:acetate mol ratios favor elongation to hexanoate or longer carboxylates whereas lower ethanol:acetate ratios mainly stall at butyrate [54, 55].

In Lucas soil microcosms with acetate + H_2_, ethanol production was recorded (Fig. S1), likely via acetate reduction with H_2_ (Eq. 3 in reverse, ${\Delta} {\mathrm{G}}^{\circ \prime } = - 9.7\,\frac{{{\mathrm{kJ}}}}{{{\mathrm{reaction}}}}$). These data allude to a requirement for an organic electron donor to initiate chain elongation of acetate to butyrate. In chain elongation, ethanol is the source for deriving the acetyl-CoA that elongates the carboxylate during a reverse β-oxidation cycle [56] and it is considered the most efficient reduced substrate for synthesis of butyrate and hexanoate in *C. kluyveri* [35]. Among enrichment conditions, concentrations of C4-C6 metabolites were lowest in the acetate + H_2_ enrichment for Lucas soil (Fig. S1). Conversion of acetate and H_2_ to ethanol (Eq. 3 in reverse) is typically characterized by sluggish kinetics [15], which likely limited the extent of butyrate and hexanoate production during the incubation time frame of the cycles (6–16 days). Butyrate production from acetate reduction with H_2_ is not a likely pathway in the microcosms and enrichments, as condensing two acetyl-CoA molecules is a highly endergonic reaction [9].

We used the time-course data from cycle 5 (shown in Fig. S4 from day 1 to 7) to determine the stoichiometry involved in chain elongation of acetate and ethanol by soil microorganisms after enrichment. To simplify the stoichiometries, we normalized all carboxylate and alcohol metabolites to butyrate mol C equivalents (e.g., 1 mol butanol = 1 mol butyrate, 1 mol hexanoate/hexanol = 1.5 mol butyrate, and 1 mol octanoate = 2 mol butyrate). We determined that the microbial communities from Tempe and Lucas soils were following the approximate stoichiometry from Eq. 4, while Bozeman soil stoichiometry was more similar to Eq. 5:

4 $$5\,{\mathrm{CH}}_3{\mathrm{CH}}_2{\mathrm{OH}} + 3\,{\mathrm{CH}}_3{\mathrm{COO}}^ - \to 4\,{\mathrm{CH}}_3{\mathrm{CH}}_2{\mathrm{CH}}_2{\mathrm{COO}}^ - \\ + \, {\mathrm{H}}^ + + 2\,{\mathrm{H}}_2 + 3\, {\mathrm{H}}_2{\mathrm{O}}$$

5 $$5\,{\mathrm{CH}}_3{\mathrm{CH}}_2{\mathrm{OH}} + 2\,{\mathrm{CH}}_3{\mathrm{COO}}^ - \to 3.5\,{\mathrm{CH}}_3{\mathrm{CH}}_2{\mathrm{CH}}_2{\mathrm{COO}}^ - \\ + \,1.5\,{\mathrm{H}}^ + + 2\,{\mathrm{H}}_2 + 2\, {\mathrm{H}}_2{\mathrm{O}}$$

The stoichiometries from our soil enrichments support previously proposed pathways requiring a ratio of 1 mol ethanol to 0.6 mol acetate consumed for every 0.8 mol butyrate produced [2].

### The prevalence and diversity of *Firmicutes* in chain-elongating soil enrichments

**Fig. 5:**
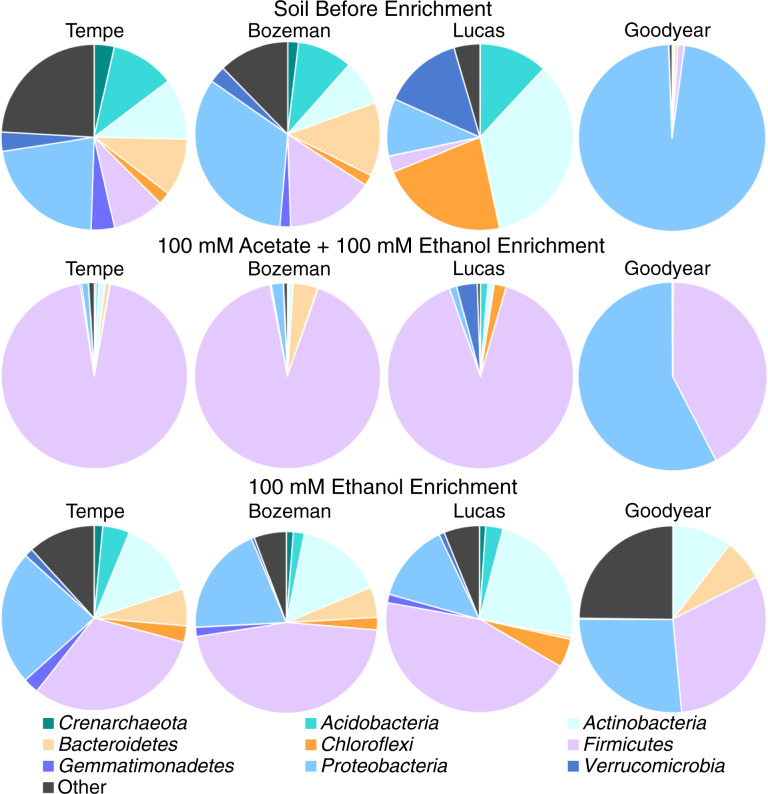
The predominance of *Firmicutes* in chain-elongating soil microbial communities enriched on acetate and ethanol or ethanol. The data are averages of sequences from duplicate microcosms before enrichment (at time zero) and after enrichment in 4 or 5 semi-batch cycles as specified in Table 2.

The initial microbial ecology at the phylum level in soils (at time zero) and the community composition in response to chain elongation enrichment (end of cycles 4 or 5) are presented in Fig. 5. Tempe and Bozeman soils sampled closest to ground surface (0.2–0.4 m) had an initial, similar community structure, abundant in sequences most similar to *Proteobacteria*, *Bacteroidetes*, and *Firmicutes* (Fig. 5) and consistent with the typical phylum distribution in top soils from various geographical regions [57, 58]. In Lucas soil, the initial microbial community was dominated by *Actinobacteria*, *Chloroflexi*, and *Verrucomicrobia* (Fig. 5). The overwhelming majority of sequences in Goodyear soil belonged to *Proteobacteria* (Fig. 5). The relative abundance of the dominant phyla in Lucas and Goodyear likely reflects some of the key soil characteristics (Table 1), including deeper depth of sampling, high-clay content and low pH (soil L), and lower organic carbon content.

Knowledge on the microbial ecology involved in chain elongation has emerged only recently and stems exclusively from biotechnology-focused studies. The enrichment of Gram-positive, spore-forming *Firmicutes* has been consistently documented in chain-elongation laboratory batch cultures and bioreactors fed with defined simple substrates or complex feedstocks [15, 53, 55, 56]. Similarly, regardless of the initial phylum distribution, we observed the predominance of *Firmicutes* in all soils and conditions that promoted chain elongation (Figs. 5 and S6). To date, only a few strains from *Clostridium* and *Eubacterium* capable of acetate and ethanol chain elongation have been isolated [2]; thus, the ability of microorganisms to grow by chain elongation in most studies with mixed microbial communities is inferred from supporting rather than direct evidence.

**Fig. 6:**
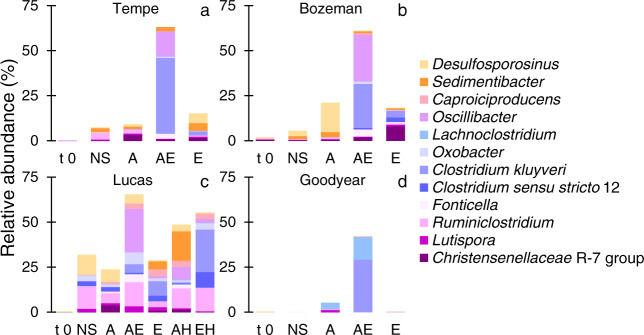
Diversity of select enriched genera and species in (a) Tempe, (b) Bozeman, (c) Lucas, and (d) Goodyear soil chain-elongating microbial communities. The plotted ASVs show relative abundance from the total sequences obtained in each condition and sampled according to Table 2. The data are averages of sequences from duplicate microcosms. Labels: t 0 = soil before enrichment; NS =  No Substrates, A = 140 mM Acetate; AE = 100 mM Acetate + 100 mM Ethanol; E = 100 mM Ethanol; AH = 100 mM Acetate + H_2_; EH = 100 mM Ethanol + H_2_.

Given the featured role of *Firmicutes* in microbial chain elongation, we further examined the identified genera and species from this phylum (Fig. 6). Miseq-based sequencing of the V4 hyper-variable region of the 16S rRNA gene has been used for species identification but is certainly less accurate than low-throughput methods sequencing the entire 16S rRNA gene [59]. Yet, we note that one identified species common across all soils substantially increased in relative abundance under all chain-elongating conditions: *C. kluyveri*, a strictly chain-elongating bacterium and the model organism for this process. ASVs most similar to *C. kluyveri* (100% match with *C. kluyveri* DSM 555) were at detectable abundances (≤0.05%) in all soils before enrichment at time zero (Fig. 6, t 0). After enrichment, *C. kluyveri* relative abundance increased to 24–42% of total sample sequences in the acetate and ethanol enrichments of Tempe, Bozeman, and Goodyear soils and to 5% in Lucas soil (Fig. 6, AE). The prevalence of *C. kluyveri* in mixed microbial communities have been recognized in chain elongation bioreactors containing acetate and ethanol [15, 16, 21], although its complete absence has also been noted under such conditions [49, 55, 60].

Beside *C. kluyveri*, the most prominent ASV in relative abundance was assigned to the genus *Oscillibacter* when using SILVA database (as described in Materials and methods section) and to *Oscillospira* when using Greengenes database v. 13_8 (data not shown). Sequences from this study most similar to *Oscillibacter* were 100% match to uncultured *Oscillibacter* sp. and 99.3% match to uncultured *Oscillospira*. At time zero in all soils, *Oscillibacter* ASVs were detected at ≤0.08% relative abundance (Fig. 6, t 0) and comprised 14–26% of the total sequences upon acetate and ethanol enrichment in Tempe, Bozeman, and Lucas soils (Fig. 6a–c, AE). In Lucas soil, *Oscillibacter* ASV was five times more abundant than *C. kluyveri* in the acetate and ethanol condition (Fig. 6c, AE). The occurrence of *Oscillibacter*- or *Oscillospira*-type microorganisms has rarely been reported or examined in soils [61, 62]. Instead, *Oscillibacter* and *Oscillospira* (type strain *Oscillospira guilliermondii* corrig. Chatton and Pérard 1913) have been of interest as perpetual microbiome members in the rumen of cattle, sheep, and other herbivores, and the human gut [61, 63–65]. Both *Oscillibacter ruminantium* and *Oscillibacter valericigenes* grow fermentatively on glucose with butyrate and valerate as main fermentation metabolites, respectively [65, 66]. No isolates of *Oscillospira* have yet been obtained. Genomic analyses from human gut samples suggest *Oscillospira* has a fermentative metabolism and produces butyrate, likely utilizing glucuronate, a sugar generated by the human host or obtained from diet containing animal products [67]. Sequences of *Oscillibacter* and/or *Oscillospira* at high relative abundance, even greater than 20% of the total sequences, have been reported in chain-elongating bioreactors converting acetate and ethanol to butyrate, hexanoate, and octanoate at pH 5.2 [49, 55], in butyrate- and hexanoate-producing bioreactors fed with synthesis gas [68], or in acetate and ethanol-fed and CO-fed batch cultures [31, 34, 56]. Nonetheless, the metabolism of these microorganisms under chain-elongating conditions remains unknown.

ASVs including *Fonticella*, *Oxobacter*, and *Ruminoclostridium* also substantially enriched relative to time zero under chain-elongating conditions (Fig. 6a–c, AE, E, AH, EH). Species of *Fonticella* ferment glucose and other sugars to ethanol and acetate [69] while *Oxobacter* sp. produce butyrate from fermentation of CO and methoxybenzenoids [70]. In the enrichments from this study, *Ruminoclostridium* ASV (but also *Oscillibacter*) was at a higher relative abundance in Lucas soil, which had an initial pH of 5.7 (Table 1). Lucas soil also maintained a mildly acid pH during enrichment on acetate and ethanol in semi-batch cycles (Fig. S5). While pH was not the only difference between soils (Table 1), the more acidic pH possibly explains why *C. kluyveri* was lowest in abundance in Lucas soil enrichment with 100 mM acetate + 100 mM ethanol (Fig. 6c, AE) compared to Tempe, Bozeman, and Goodyear counterparts. Strain K1 of *C. kluyveri* (DSM 555) isolated from canal mud grows at pH 6–7.5, with an optimal pH of 6.8 [1]. Strain 3231B of *C. kluyveri* isolated from bovine rumen grows in a wider range of pH (4.9–9.2), although its maximum growth rate is achieved at pH 7.6 [4]. A strain from *Ruminococcaceae* (CPB6), most similar to the hexanoate-producing *Clostridium* sp. BS-1 [71], has been isolated and is capable of chain elongation of lactate to butyrate and hexanoate at pH 5–6.5 [72]. Nonetheless, with the exception of *C. kluyveri*, the enriched ASVs from Fig. 6 have not been reported to grow by conversion of acetate and ethanol to C4-C8 carboxylates or by reduction of C4-C6 carboxylates with H_2_ to C4-C6 alcohols (Figs. 2–3). The soil enrichments also provided conditions for growth of microorganisms with butyrate- and ethanol-consuming capabilities, including *Desulfosporosinus* [73, 74]. Species of *Desulfosporosinus* have been co-enriched during bioreactor chain elongation [49, 55] but *Desulfosporosinus* relative abundance has been negatively correlated with production of more elongated carboxylates.

### A potential niche for microbial chain elongation in soils

We have not yet examined chain elongation in situ nor do we know the extent to which this process occurs under natural conditions. Given the artificial conditions in laboratory incubations, the concentrations of microbial chain elongation metabolites observed in our microcosms are not likely to be typical of in situ concentrations. Still, on the basis of this study and our understanding of the process, we expect chain elongation to be triggered during soil events of high release of carboxylates and reduced electron donors (ethanol, H_2_) from animal, plant, and microbial decay. Methane production was purposely inhibited in our experiments to exalt chain-elongation activity. However, methanogenesis can co-occur with chain-elongation [1]. In biotechnology-based research, losses of chain elongation substrates to methane are purposely managed by adding methanogenesis-specific inhibitors such as BES [15] or by selecting bioreactor operating conditions that minimize growth of methanogens (e.g., low pH, high dilution rate) [2]. Conditions that promote chain elongation versus other metabolic processes utilizing simple fermentation products including methanogenesis are probably a combination of several environmental factors. At this point, we can postulate that microbial chain elongation may serve a mechanism to bank electrons and carbon from labile substrates into higher-carbon compounds and to overall delay losses of organic compounds through acetoclastic methanogenesis or mineralization within a soil.

## Conclusions

Chain elongation of acetate and ethanol to butyrate and hexanoate as a growth-dependent metabolism was first described in the soil bacterium, *C. kluyveri*, more than eight decades ago. Current efforts using complex microbial communities have propelled to fame chain elongation as a biotechnological platform for production of medium-chain carboxylates and other biochemicals. Nonetheless, the possibility of chain elongation as a soil process has remained obscure. This study is the first directed examination on microbial chain elongation as a metabolism involving carbonaceous substrates under anaerobic conditions in soils. We demonstrated that different soil types with various characteristics harbor a readily active potential for chain elongation of acetate and ethanol, typical metabolites from organic compound fermentation. Chain elongation was easily exalted by the presence of acetate and ethanol at concentrations much higher than naturally occurring in soils but also at environmentally relevant concentrations of these substrates. Microorganisms most similar to *C. kluyveri* became highly enriched under chain-elongating conditions. The co-enrichment of several other *Firmicutes* genera suggests this metabolism may extend to other soil microorganisms. Results from this study support that chain elongation as an energy-conserving pathway should not be overlooked in soil microbial ecology studies. That chain elongation can occur in soils is maybe not surprising but its dynamics in situ is unclear. The extent and role of microbial chain elongation under natural soil conditions is a pertinent research focus in our group.

## Supplementary information

Supplementary information
