# Supplementary material for: The occurrence and ecology of microbial chain elongation of carboxylates in soils
Source: ISME J. 2021 Feb 8;15(7):1907–18. doi: 10.1038/s41396-021-00893-2 (PMC8245554; doi:10.1038/s41396-021-00893-2)
Supplement: Supplementary file 1 — Supplementary information [file 41396_2021_893_MOESM1_ESM.pdf]

**The occurrence and ecology of microbial chain elongation of carboxylates in soils**

Sayalee Joshi, Aide Robles, Samuel Aguiar, and Anca G. Delgado

**Supplementary Information**

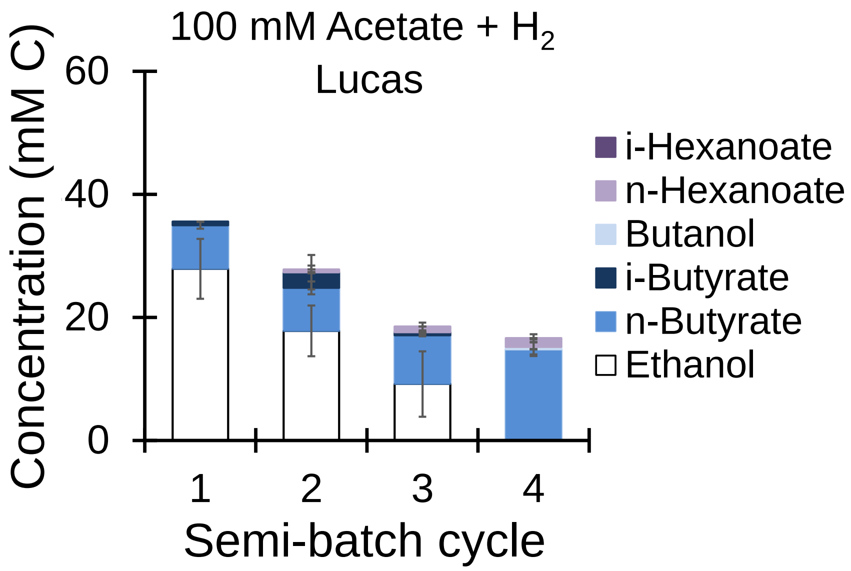


**Figure S1**. Metabolites during microbial chain elongation in soils initially fed with 100 mM acetate and 90 mM $H_2$ and subjected to semi-batch enrichment. A semi-batch cycle consisted of removing one third of microcosm liquid (25 mL) and replacing with 25 mL medium containing 100 mM acetate. The incubation time for each cycle (between 6-16 days) is shown in Table 2 in the main text. The plotted carboxylates and alcohols are final metabolite concentrations at the end of each cycle before liquid was removed and medium with substrates was readded. The data are averages with standard deviation of duplicate microcosms.

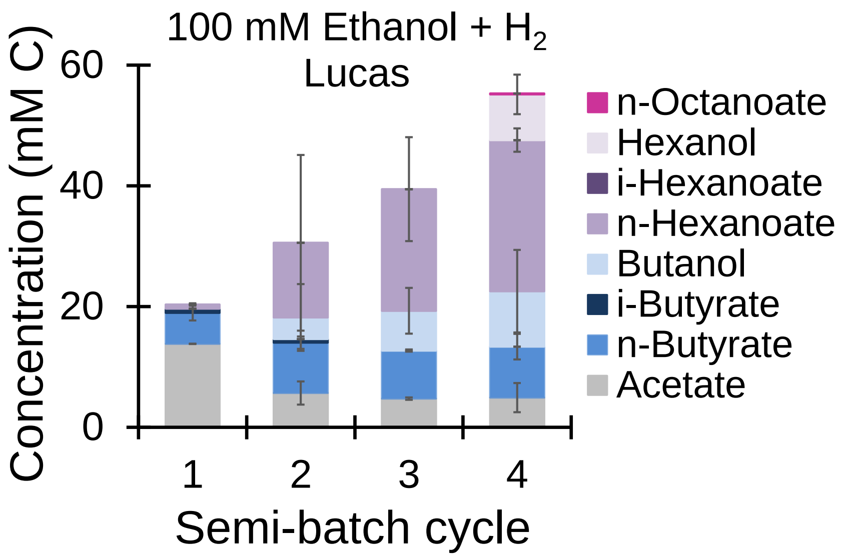


**Figure S2**. Metabolites during microbial chain elongation in soils initially fed with 100 mM ethanol and 90 mM $H_2$ and subjected to semi-batch enrichment. A semi-batch cycle consisted of removing one third of microcosm liquid (25 mL) and replacing with 25 mL medium containing 100 mM ethanol. The incubation time for each cycle (between 6-16 days) is shown in Table 2 in the main text. The plotted carboxylates and alcohols are final metabolite concentrations at the end of each cycle before liquid was removed and medium with substrates was readded. The data are averages with standard deviation of duplicate microcosms.

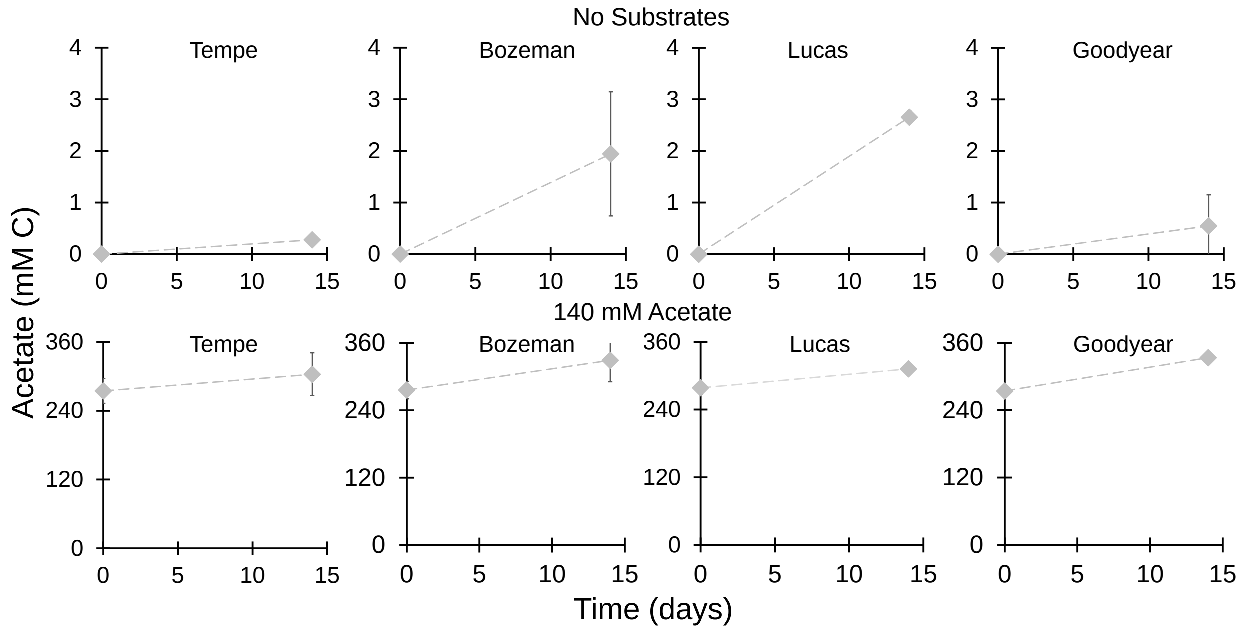


**Figure S3**. (Top panels) Acetate production in soil microcosms in the absence of added substrates. (Bottom panels) Absence of microbial chain elongation metabolites in soil microcosms with 140 mM acetate as the sole added substrate. The data are averages with standard deviation of duplicate microcosms.

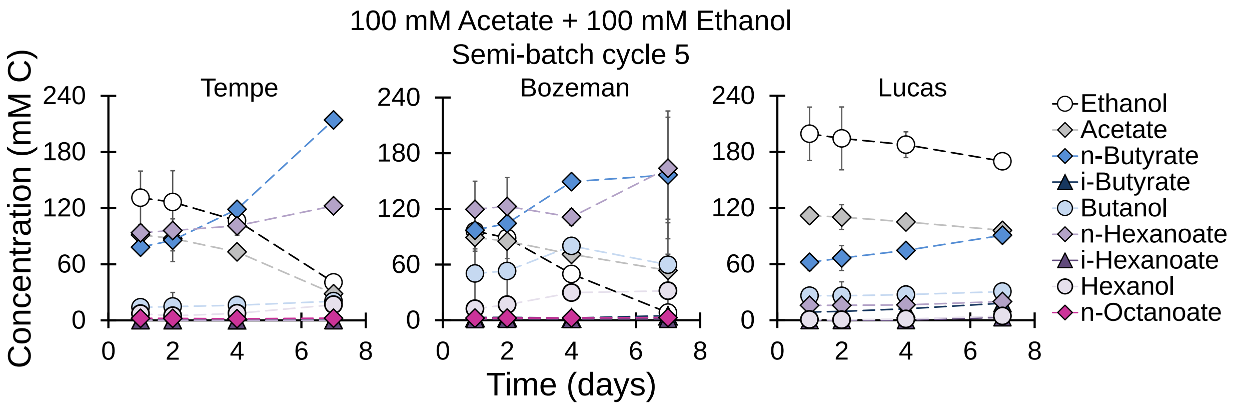


**Figure S4**. Conversion of acetate and ethanol to microbial chain elongation metabolites during semi-batch cycle 5. The data are averages with standard deviation of duplicate microcosms.

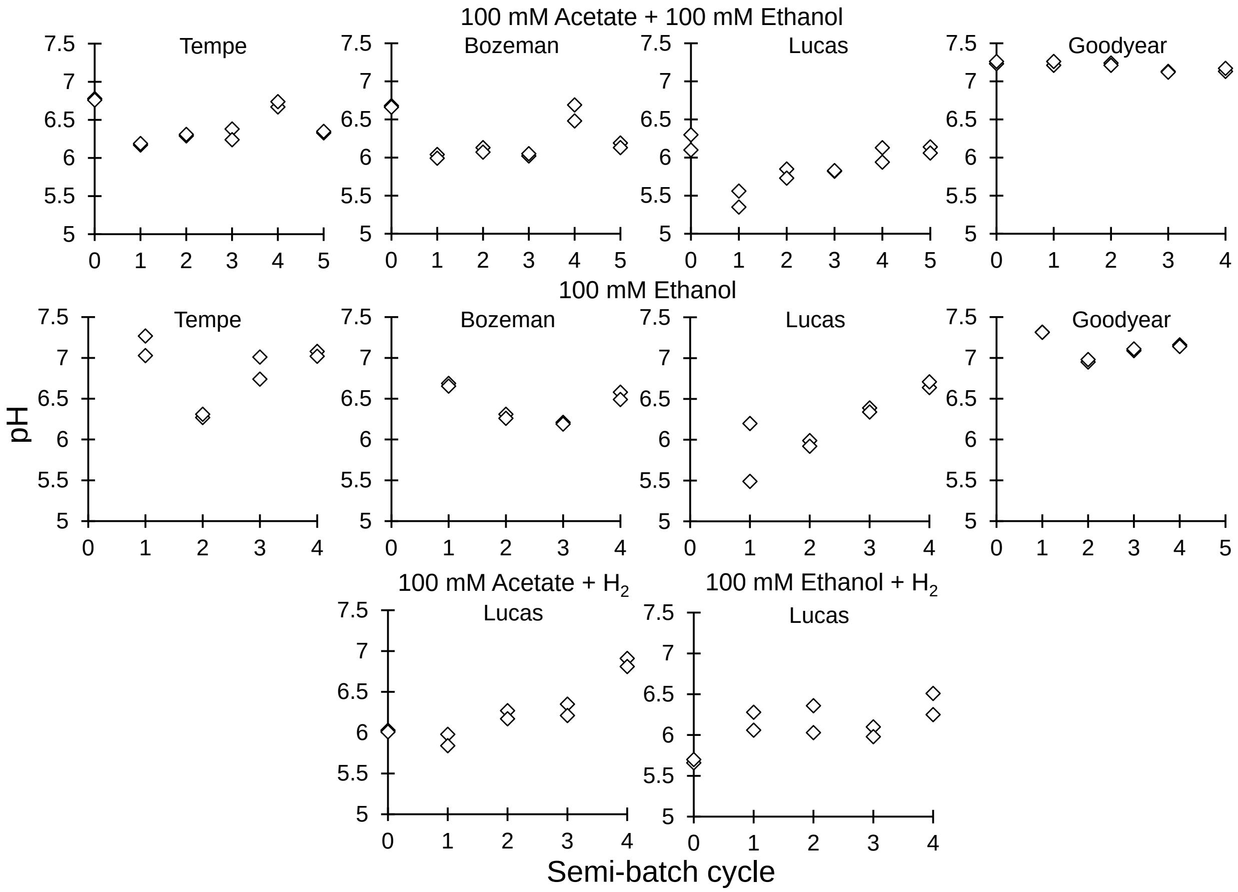


**Figure S5.** pH measurements in replicate microcosms subjected to enrichment in semi-batch cycles.

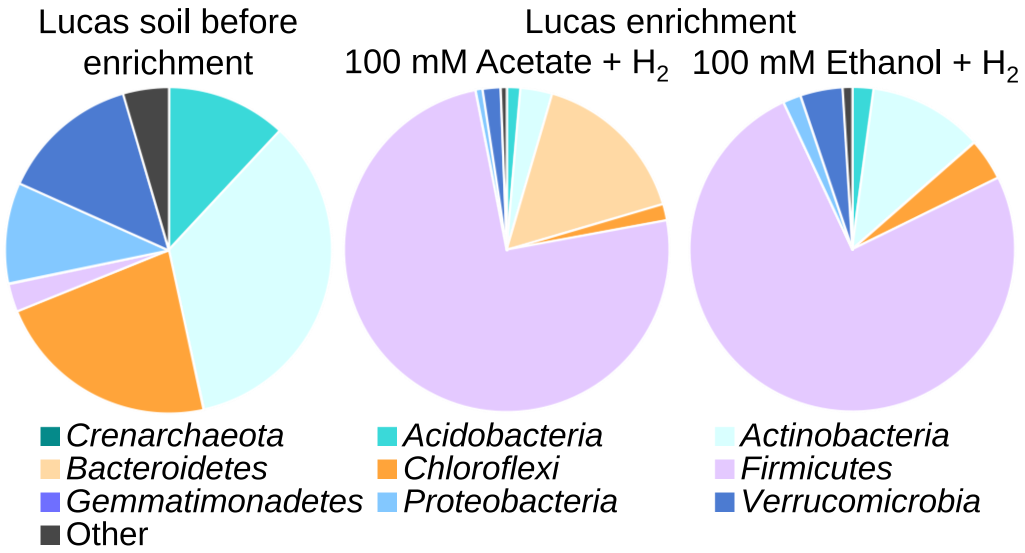


**Figure S6**. Microbial community composition at the phylum level in Lucas soil at time zero and after enrichment on acetate/ethanol and $H_2$ after 4 semi-batch cycles. The data are averages of sequences from duplicate microcosms.

**Table S1.** Carbon (C) and electron balances at the end of cycle 1 (day 14) in the Tempe, Bozeman, Lucas, and Goodyear soil microcosms with 100 mM acetate and 100 mM ethanol or 100 mM ethanol. The data are percent millielectron equivalents (me$^{-}$ equiv.) or mmol C recovered as metabolites from the consumed substrate(s) (acetate and ethanol or ethanol). For these calculations, the substrate(s) and metabolites were converted to me$^{-}$ equiv. or mmol C according to their stoichiometries described in the Materials and Methods.

| **Cycle 1** | **100 mM Acetate + 100 mM Ethanol** | | | | **100 mM Ethanol** | | | |
|---|---|---|---|---|---|---|---|---|
| **Metabolite** | Tempe | Bozeman | Lucas | Goodyear | Tempe | Bozeman | Lucas | Goodyear |
| Acetate (%) | – | – | – | – | 4 | 0.4 | 0.2 | 0 |
| Butyrate (%) | 55 | 53 | 38 | 19 | 0 | 4 | 3 | 0 |
| Butanol (%) | 14 | 14 | 14 | 0 | 0 | 0 | 0.1 | 0 |
| Hexanoate (%) | 35 | 29 | 1 | 0 | 0 | 27 | 21 | 0 |
| Hexanol (%) | 0 | 0 | 0 | 0 | 0 | 0 | 0 | 0 |
| Octanoate (%) | 0 | 0 | 0 | 0 | 0 | 0 | 0 | 0 |
| Total me$^{-}$ equiv. (%) | 104 | 96 | 53 | 19 | 4 | 32 | 24 | 0 |
| Total C (%) | 117 | 99 | 58 | 16 | 6 | 36 | 28 | 0 |
